# Re‐appraisal of the universal definition of tumor rupture among patients with high‐risk gastrointestinal stromal tumors

**DOI:** 10.1002/ags3.12684

**Published:** 2023-04-26

**Authors:** Naoto Gotohda, Toshirou Nishida, Shinsuke Sato, Masato Ozaka, Yujiro Nakahara, Yoshito Komatsu, Masato Kondo, Haruhiko Cho, Yukinori Kurokawa, Yuko Kitagawa

**Affiliations:** ^1^ Department of Hepatobiliary and Pancreatic Surgery National Cancer Center Hospital East Kashiwa Japan; ^2^ Department of Surgery National Cancer Center Hospital Tokyo Japan; ^3^ Department of Gastroenterological Surgery Shizuoka General Hospital Shizuoka Japan; ^4^ Department of Hepato‐Biliary‐Pancreatic Medicine, Gastroenterology Center Cancer Institute Hospital Japanese Foundation for Cancer Research Tokyo Japan; ^5^ Department of Gastroenterological Surgery Osaka Police Hospital Osaka Japan; ^6^ Department of Gastroenterology and Hepatology Hokkaido University Hospital Hokkaido Japan; ^7^ Department of Surgery Kobe City Medical Center General Hospital Kobe Japan; ^8^ Department of Surgery Tokyo Metropolitan Cancer and Infectious Diseases Center Komagome Hospital Tokyo Japan; ^9^ Department of Gastroenterological Surgery Osaka University Graduate School of Medicine Osaka Japan; ^10^ Department of Surgery Keio University Hospital Tokyo Japan

**Keywords:** gastrointestinal stromal tumor, high‐risk GIST, tumor rupture, universal definition

## Abstract

**Aim:**

Tumor rupture has been indicated as a risk factor for recurrence of gastrointestinal stromal tumors (GISTs). The universal definition of tumor rupture was proposed. This study evaluated whether the universal definition was more accurate in identification of GISTs with high recurrent risk than subjective judgment.

**Methods:**

The study included 507 patients with high‐risk GISTs who underwent complete resection between December 2012 and December 2015. We conducted a questionnaire survey in participating institutes to re‐diagnose tumor rupture based on the universal definition according to their surgical and pathological findings. We compared the clinical outcomes of tumor rupture based on the definition to those based on the surgeon's judgment and clarified the clinical importance of the rupture.

**Results:**

Sixty‐four patients were initially registered to have tumor rupture by surgeon's judgment, and it became 90 patients who had tumor rupture after reevaluation. Although there were significant differences in recurrence‐free survival (RFS) between no rupture and rupture for both initial registration and reevaluation (*p* = 0.002, <0.001, respectively), a significant difference in overall survival was only observed after reevaluation (*p* = 0.011). Tumor rupture was significantly associated with large tumor size, mixed cell type in histology, R1 resection, frequent adjuvant therapy and recurrence, but not with location, mitosis, and genotype. Adjuvant therapy more than 3 years improved RFS of patients with tumor rupture.

**Conclusion:**

This study suggested that tumor rupture based on the universal definition more accurately identified GISTs with poor prognostic outcomes than the subjective judgment.

## Graphical abstract

Overall survival (OS) was not different between non‐tumor rupture (NTR) and tumor rupture (TR) groups in the initial report (*p* = 0.210). The 3‐year/5‐year OS rates of NTR and TR groups were 97.9/93.1% (NTR, 3y/5y) and 96.7/85.4% (TR, 3y/5y), respectively. In contrast, OS was significantly different between the two groups in the reevaluated report (*p* = 0.011).

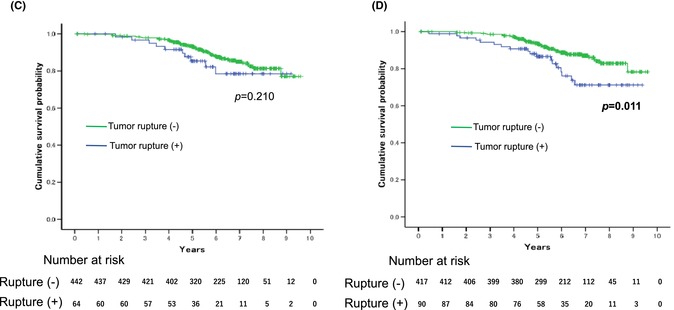

## 1 INTRODUCTION

Gastrointestinal stromal tumor (GIST) is the most frequent sarcoma in the gastrointestinal tract. Complete resection is the gold standard in the treatment of primary GISTs. Even after complete resection, approximately 40% of GIST patients may have recurrence during follow‐up.^1^, ^2^, ^3^ Tumor size, mitosis count, tumor site, and tumor rupture are considered to be risk factors for recurrence.^1^, ^4^, ^5^, ^6^, ^7^, ^8^, ^9^ Among the four factors, tumor size, mitosis count, and tumor site are documented objectively even in retrospective previous studies, and are included in the most risk stratification and nomograms. The prognosis of patients with ruptured GISTs was reported as poor,^5^, ^8^, ^10^, ^11^, ^12^, ^13^ however, as the diagnosis of tumor rupture is clinical judgment and is infrequent, prognostic outcomes of patients with ruptured GISTs were inconsistent. Clinical significance of tumor rupture is still controversial due to the lack of a commonly shared definition and low statistical power. Therefore, tumor rupture is not always included in the risk stratification or nomograms.

It has been shown that surgeons have varied criteria in their judgment of tumor rupture.^14^ Therefore, the universal definition of tumor rupture in GIST was proposed in 2019.^15^ This observational substudy on patients from the prospective registry study (the STAR ReGISTry study^16^) evaluated whether a clinical decision based on the universal definition more efficiently identifies GISTs with high recurrent risk compared with individual judgment. We also examined prognostic outcomes of ruptured GISTs and evaluated the clinical significance of tumor rupture based on the universal definition.

## 2 PATIENTS AND METHODS

### 2.1 Patients

A total of 541 patients with primary high‐risk GISTs were enrolled to the prospective observational registry study (the STAR ReGISTry study) after complete resection (R0 or R1) between December 2012 and December 2015.^17^ The modified NIH consensus criteria were adopted for the risk stratification.^1^ Other inclusion criteria include *KIT*‐positive or mutation‐positive in *KIT*, *PDGFRA*, and/or other genes, no metastatic disease (neither metastasis nor peritoneal disease), older than 20 years of age, and written informed consent. Patients with other cancers, cardiovascular, brain, hepatic, or renal diseases and recurrent diseases were excluded. Surgical specimens were confirmed by the central pathological diagnosis.

Among 541 patients, 26 patients were excluded for various reasons including unmet inclusion criteria and non‐GISTs by the central pathological diagnosis and, finally, 515 patients were evaluated in the STAR ReGISTry study.^16^ This study also evaluated the same 515 patients. The clinicopathological data of registered patients were collected from participating institutions. The tumor rupture was judged by a physician or a surgeon of each institution in the initial registry. However, the definition of tumor rupture was proposed in 2019.^15^ Thus, all registered patients were reevaluated according to the classification of the universal definition of tumor rupture. The data of eight patients were not available in terms of the rupture classification and were excluded from the study. Finally, 507 GIST patients with or without tumor rupture were retrospectively analyzed. The treatment for GIST patients was not prescribed in the protocol, however, the adoption of the Clinical Practice Guidelines 2012^18^ was recommended among participating institutions.

## 3 METHODS

This study is an observational study as the STAR ReGISTry study.^16^ After identifying cases of tumor rupture registered in the STAR ReGISTry study, a questionnaire was sent to all participating institutions to reevaluate enrolled patients based on surgical and pathological findings based on the universal definition. The subtypes of tumor rupture were also requested to classify according to the definition in this questionnaire. We compared the clinical outcomes of tumor rupture based on the universal definition with those based on the surgeon's initial judgment at registry. Additionally, we evaluated prognostic effects of adjuvant therapy according to treatment duration: less than 1 year, between 1 year and 3 years, and more than 3 years.

The study was conducted in accordance with the World Medical Association Declaration of Helsinki, Ethical Principles for Medical Research Involving Human Subjects (Amended in Seoul in October 2008), and the Ethics Guidelines for Clinical Research (Ministry of Health, Labour and Welfare Notice No. 415, 2008). Ethical approval was initially obtained from the institutional review board (IRB) of the National Cancer Center and then from the IRBs of the other participating hospitals (No. 2016–250). Written informed consent was obtained from all participating patients. The STAR ReGISTry study was registered with the UMIN Clinical Trials Registry, number UMIN000009531.

### 3.1 The classification of tumor rupture in GIST

The classification of tumor rupture in GIST was proposed by Nishida et al in 2019.^15^ The spectrum of tumor rupture in GIST was represented in six different clinical scenarios. The classification was the following six definitions for “tumor rupture”: (type 1) tumor fracture or spillage; (type 2) blood‐stained ascites; (type 3) gastrointestinal perforation at the tumor site; (type 4) microscopic infiltration of an adjacent organ; (type 5) piecemeal resection or intralesional dissection; or (type 6) incisional biopsy. Furthermore, tumor fracture is classified as type 1A and tumor spillage is classified as type 1B.

### 3.2 Statistical analysis

Recurrence‐free survival (RFS) and overall survival (OS) of this study were defined as the same as in the STAR ReGISTry study. RFS was defined as the time from the date of surgery to the first documented recurrence or death from any cause. OS was defined as the time from the date of surgery to the death from any cause.

Categorical variables were tabulated using *n* (%) and continuous variables using median and interquartile range (IQR). For comparisons of categorical variables, a chi‐squared test was used, and for comparisons of continuous variables, a Mann–Whitney U test was used. The cumulative survival rates for RFS and OS were estimated by the Kaplan–Meier method. The hazard ratio (HR) and its 95% confidence interval (95%CI) were calculated using the Cox's proportional hazards model. The *p* values were two‐sided, and *p* values <0.05 were considered significant. All statistical analysis were performed using SPSS PASW version 18.0 (SPSS Inc.).

## 4 RESULTS

### 4.1 Changes in tumor rupture after definition

Among 507 patients, 64 patients were registered to have tumor rupture in the initial case report of the STAR ReGISTry study, and 90 patients were reported to have tumor rupture by the questionnaire.

Changes in subtypes of tumor rupture are shown in Table 1. The total number of subtypes of tumor ruptured patients was 74 (some patients had multi‐subtypes) in the initial report. Eleven patients with blood‐stained ascites (type 2), 14 with adjacent organ infiltration (type 4), one with piecemeal resection/intralesional dissection (type 5), and three with incisional biopsy (type 6) were added as ruptured GIST patients by the reevaluation (some overlapping). Of 90 patients, 17 patients had multiple factors. One patient was type 1A and 2 and 3 with three types of overlap, and the remaining 16 had two factors of overlap: seven with types 1A and 2, one with types 1B and 2, one with types 1B and 3, two with types 2 and 4, two with types 2 and 5, two with types 3 and 4, and one with types 4 and 5. Comparisons between each group were difficult to analyze due to the small number of cases.

**TABLE 1:** The distribution of tumor rupture patients based on the defined classification.

The defined classification of tumor rupture	Number of initial report of tumor rupture	Number of reevaluated report of tumor rupture based on defined classification
Tumor fracture (type 1A)	21	21
Tumor spillage (type 1B)	5	5
Blood‐stained ascites (type 2)	19	30
Gastrointestinal perforation on tumor (type 3)	9	9
Adjacent organ infiltration (type 4)	7	21
Piecemeal resection or intralesional dissection (type 5)	13	14
Incisional biopsy (type 6)	0	3
Total	74	103

### 4.2 Clinicopathological characteristics of patients with tumor rupture

Among 507 registered patients, we compared clinicopathological features of patients without tumor rupture (NTR group; *N* = 417) and those with the rupture (TR group; *N* = 90) according to the universal definition. Between NTR and TR groups, there were no significant differences in age, gender, performance status (PS), tumor location (stomach, small intestine and rectum, or others), mitosis, and genotype (Table 2). Microscopically residual tumor (R1) was significantly higher in the TR group compared with the NTR group (TR and NTR: 14.4% vs. 1.0%, *p* < 0.001). Tumor size was significantly larger in the TR group (median size of TR and NTR: 10 and 7 cm, *p* < 0.001), and, thus, open surgery was more frequent in the TR group (TR and NTR: 84.4 vs. 69.3%, *p* = 0.002).

**TABLE 2:** Patients' clinicopathological characteristics according to the status of tumor rupture.

	Non‐tumor rupture group (*N* = 417)	Tumor rupture group (*N* = 90)	*p* value
Age (median, IQR; years)	64 (56–72)	64 (55–69)	0.343
Gender			
Male / Female	224 (53.7%) / 193 (46.3%)	51 (56.7%) / 39 (43.3%)	0.348
Performance status			
0 / 1–3	351 (84.8%) / 63 (15.2%)	76 (84.4%) / 14 (15.6%)	0.523
Location			
Stomach	256 (61.4%)	52 (57.8%)	0.600
Small intestine and Rectum	152 (36.5%)	36 (40%)	
Others	9 (2.2%)	2 (2.2%)	
Curability of surgery			
R0 / R1	413 (99.0%) / 4 (1.0%)	77 (85.6%) / 13 (14.4%)	**<0.001**
Surgical approach			
Open / Laparoscopic surgery	289 (69.3%) / 128 (30.7%)	76 (84.4%) / 14 (15.6%)	**0.002**
Tumor size (median, IQR; cm)	7 (5.3–11)	10 (6.5–14)	**<0.001**
Recurrence (Y/N)	124 (29.7%) / 293 (70.3%)	47 (52.2%) / 43 (47.8%)	**<0.001**
Recurrence site			
Liver	68 (55.3%)	18 (38.3%)	**0.035**
Peritoneum	52 (42.3%)	30 (63.8%)	**0.009**
Local site	20 (16.3%)	3 (6.4%)	0.070
Others	9 (7.3%)	3 (6.4%)	0.566
Neoadjuvant therapy (Y/N)	48 (11.5%) / 369 (88.5%)	9 (10.0%) / 81 (90.0%)	0.422
Adjuvant therapy (Y/N)	329 (78.9%) / 88 (21.1%)	79 (87.8%) / 11 (12.2%)	**0.033**
Duration of adjuvant (median, IQR; years)	3.0 (1.0–3.8)	2.5 (0.7–3.0)	**0.023**
Mitosis (median, IQR; /50HPF)	5 (1–20)	5 (1–19)	0.790
Unavailable	4	1	
Histological type			
Spindle	362 (90.3%)	63 (75.0%)	**<0.0001**
Epithelioid	8 (2.0%)	7 (8.3%)	
Mixed	31 (7.7%)	14 (16.7%)	
Unavailable	16 (3.8%)	6 (6.7%)	
Genotyping			
*KIT*	371 (93.0%)	80 (90.9%)	0.548
*PDGFRA*	13 (3.3%)	5 (5.7%)	
Wild type	15 (3.8%)	3 (3.4%)	
Unavailable	18 (4.3%)	2 (2.2%)	

Bold indicates the *p* values less than 0.05 were considered significant.

Although the number of patients who underwent neoadjuvant therapy was similar between the two groups, patients with adjuvant therapy was significantly higher in the TR group (TR and NTR: 87.8 vs. 78.9%, *p* = 0.033). The duration of adjuvant therapy was significantly shorter in the TR group (median duration of TR and NTR: 2.5 vs. 3.0 years, *p* = 0.023). In the histological cell type, mixed (spindle and epithelioid) features are more predominant in the TR group (16.7%) compared with the NTR group (7.7%).

Recurrence was significantly frequent in the TR group (TR and NTR: 52.2 vs. 29.7%, *p* < 0.001). The liver was the most frequent site in the NTR group (55.3%) as the first site of recurrence, the peritoneum was the most frequent site in the TR group (63.8%, Table 2).

### 4.3 Comparison of RFS and OS between NTR group and TR group

We evaluated prognostic outcomes of patients with ruptured GISTs before and after reevaluation.

**FIGURE 1:**
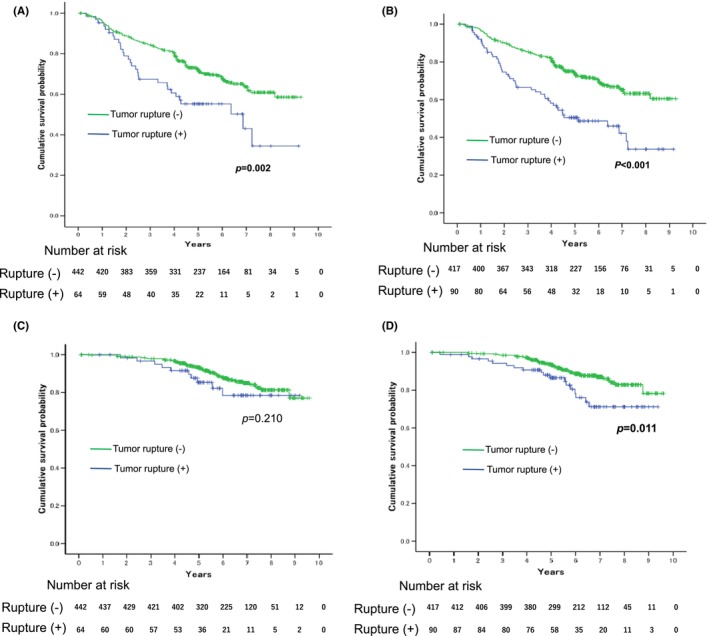
RFS and OS after surgery according to the status of tumor rupture. A and B show the RFS curves. C and D show the OS curves. A and C include 64 patients of tumor rupture group at the initial report. B and D include 90 patients of the reevaluated report of tumor rupture based on the universal definition. The green line indicates the non‐tumor rupture group, and the blue line indicates the tumor rupture group.

With the median follow‐up of 5.9 (Interquartile range [IQR] 4.8–7.0) years, RFSs were significantly different between NTR and TR groups both in the initial and reevaluated reports (*p* = 0.002, <0.001, respectively, Figure 1A, B). In the initial reports, the 3‐year/5‐year RFS rates of NTR and TR groups were 84.3/72.2% (NTR, 3y/5y) and 67.4/55.2% (TR, 3y/5y), respectively. In the reevaluated reports, the 3‐year/5‐year RFS rates of NTR and TR groups were 85.4/74.1% (NTR, 3y/5y) and 66.5/50.5% (TR, 3y/5y), respectively.

OS was not different between NTR and TR groups in the initial report (*p* = 0.210). The 3‐year/5‐year OS rates of NTR and TR groups were 97.9/93.1% (NTR, 3y/5y) and 96.7/85.4% (TR, 3y/5y), respectively. In contrast, OS was significantly different between the two groups in the reevaluated report (*p* = 0.011, Figure 1C, D). The 3‐year/5‐year OS rates of NTR and TR groups were 98.5/93.4% (NTR, 3y/5y) and 94.2/86.6% (TR, 3y/5y), respectively.

The median RFSs (95%CI) of the TR group in the initial and reevaluated reports were 6.9 (3.0–10.7) and 5.1 (3.0–7.3) years, respectively. Median OSs could not be estimated in both situations.

### 4.4 Effects of various duration of adjuvant therapy on ruptured GISTs

RFS and OS were evaluated according to adjuvant duration among 90 patients with tumor rupture based on the universal definition. Clinicopathological comparison of these patients according to various adjuvant therapy durations was shown in Table 3. No significant differences were found among the three groups. Median duration of adjuvant imatinib therapy for less than 1 year and that for between 1 and 3 years were 0.41 (IQR: 0.085–0.72) years and 2.7 (IQR: 1.9–2.9) years, respectively, and that of adjuvant therapy more than 3 years was estimated to be 3.3 (IQR: 3.0–5.0) years and five patients were still on the therapy at data cut‐off.

**TABLE 3:** Patients' clinicopathological characteristics according to various adjuvant therapy duration.

	Less than 1 year group (*N* = 37)	1 to 3 years group (*N* = 31)	More than 3 years group (*N* = 22)	*p* value
Age (median, IQR; years)	66 (56–75)	63 (54–67)	63 (56–68)	0.536
Gender				
Male / Female	16 (43.2%) / 21 (56.8%)	19 (61.3%) / 12 (38.7%)	16 (72.7%) / 6 (27.3%)	0.071
Performance status				
0 / 1–3	32 (86.5%) / 5 (13.5%)	27 (87.1%) / 4 (12.9%)	17 (77.3%) / 5 (22.7%)	0.564
Location				
Stomach / Others	21 (56.8%) / 16 (43.2%)	10 (32.3%) / 21 (67.7%)	10 (45.5%) / 12 (54.5%)	0.130
Curability of surgery				
R0 / R1	30 (81.1%) / 7 (18.9%)	27 (87.1%) / 4 (12.9%)	20 (90.9%) / 2 (9.1%)	0.557
Surgical approach				
Open / Laparoscopic surgery	28 (75.7%) / 9 (24.3%)	27 (87.1%) / 4 (12.9%)	21 (95.5%) / 1 (4.5%)	0.113
Tumor size (median, IQR; cm)	8.5 (6.4–13)	12 (6.8–17)	10 (6.4–16)	0.402
Recurrence (Y/N)	20 (54.1%) / 17 (45.9%)	19 (61.3%) / 12 (38.7%)	8 (36.4%) / 14 (63.6%)	0.193
Recurrence site				
Liver	10 (50.0%)	6 (31.6%)	2 (25.0%)	0.346
Peritoneum	12 (60.0%)	13 (68.4%)	5 (62.5%)	0.858
Local site	2 (10.0%)	1 (5.3%)	0	0.600
Others	0	2 (10.5%)	1 (12.5%)	0.300
Neoadjuvant therapy (Y)	3 (8.1%)	4 (12.9%)	2 (9.1%)	0.795
Duration of adjuvant (median, IQR; years)	0.41(0.085–0.72)	2.7 (1.9–2.9)	3.3 (3.0–5.0)	**<0.0001**
Mitosis (median, IQR; /50HPF)	4 (1–19)	4 (1–26)	8.5 (1–22)	0.848
Unavailable	0	1	0	
Histological type				
Spindle	26 (70.3%)	20 (64.5%)	17 (77.3%)	0.883
Epithelioid	3 (8.1%)	2 (6.5%)	2 (9.1%)	
Mixed	5 (13.5%)	7 (22.6%)	2 (9.1%)	
Unavailable	3 (8.1%)	2 (6.5%)	1 (4.5%)	
Genotyping				
*KIT*	29 (78.4%)	30 (96.8%)	21 (95.5%)	0.116
*PDGFRA*	5 (13.5%)	0	0	
Wild type	2 (5.4%)	0	1 (4.5%)	
Unavailable	1 (2.7%)	1 (3.2%)	0	

Bold indicates the *p* values less than 0.05 were considered significant.

**FIGURE 2:**
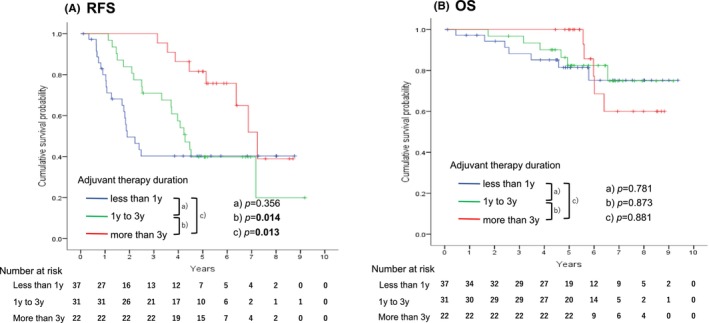
RFS and OS after surgery according to various adjuvant therapy duration among 90 patients of reevaluated report of tumor rupture based on the universal definition. A shows the RFS curves and B shows the OS curves. The red line indicates the more than 3‐year adjuvant group, the green line indicates the 1‐year to 3‐year adjuvant group and the blue line indicates the less than 1‐year adjuvant group.

In RFS, there was no difference between patients with adjuvant therapy less than 1 year and those with adjuvant for 1 to 3 years (*p* = 0.356); however, there were significant differences between patients with adjuvant for 1 to 3 years and those with adjuvant for more than 3 years (*p* = 0.014) and between less than 1 year and more than 3 years (*p* = 0.013, Figure 2A). The median RFS (95%CI) and the 3‐year/5‐year RFS rates of patients with adjuvant therapy of less than 1 year, 1 to 3 years, and more than 3 years were 1.9 (1.1–2.7) years and 40.3/40.3%, 4.3 (3.6–4.9) years and 71.0/39.8% and 7.2 (6.2–8.3) years and 100.0/81.6%, respectively.

OS was similar among three groups of adjuvant duration (less than 1 year, 1 to 3 years, and more than 3 years, Figure 2B). The 3‐year/5‐year OS rates of patients with adjuvant therapy of less than 1 year, 1 to 3 years, and more than 3 years were 88.2/81.5%, 96.8/82.4%, and 100.0/100.0%, respectively. The median OS was not estimated in all groups.

There were 16 patients who relapsed during adjuvant therapy. RFS and OS were evaluated excluding these patients (Figure S1), the results of RFS and OS were similar to Figure 2A, B.

### 4.5 Prognostic factors of OS and RFS

Prognostic analysis of Cox proportional hazards model for RFS and OS after resection of GISTs is shown in Tables 4 and 5. Univariate Cox proportional hazards model analysis revealed that tumor location, tumor size, tumor rupture, mitosis, histological type, and duration of adjuvant therapy were significantly associated with RFS. Age, performance status, tumor size, tumor rupture, mitosis, genotyping, histological type, and duration of adjuvant therapy were significantly associated with OS.

**TABLE 4:** Univariate and multivariate analysis of Cox proportional hazards model for RFS after surgery.

	No. of cases	Univariate analysis			Multivariate analysis		
		Hazard ratio	95%CI	*p* value	Hazard ratio	95%CI	*p* value
Age (years)							
≦65	280	Ref					
>65	235	1.12	0.83–1.51	0.445			
Gender							
Male	280	Ref					
Female	235	0.91	0.67–1.22	0.523			
Performance status							
0	432	Ref					
1–3	80	1.18	0.79–1.76	0.416			
Location							
Stomach	313	Ref					
Others	202	1.54	1.15–2.08	**0.004**	1.96	1.41–2.73	**<0.001**
Tumor size (cm)							
≦10.0	350	Ref					
>10.0	164	1.59	1.18–2.15	**0.003**	1.87	1.34–2.60	**<0.001**
Tumor rupture							
No	417	Ref					
Yes	90	2.25	1.61–3.15	**<0.001**	1.40	0.945–2.06	0.094
Mitosis (/50HPF)							
≦5	271	Ref					
>5	241	3.03	2.20–4.18	**<0.001**	4.19	2.95–5.94	**<0.001**
Genotyping							
KIT	457	Ref					
Others	36	0.91	0.48–1.73	0.780			
Histological type							
Spindle	433	Ref					
Epithelioid & Mixed	60	2.72	1.89–3.93	**<0.001**	2.04	1.38–3.00	**<0.001**
Duration of adjuvant (years)							
≦3	366	Ref					
>3	149	0.43	0.30–0.63	**<0.001**	0.34	0.23–0.50	**<0.001**

Bold indicates the *p* values less than 0.05 were considered significant.

**TABLE 5:** Univariate and multivariate analysis of Cox proportional hazards model for OS after surgery.

	No. of cases	Univariate analysis			Multivariate analysis		
		Hazard ratio	95%CI	*p* value	Hazard ratio	95%CI	*p* value
Age (years)							
≦65	280	Ref					
>65	235	3.34	1.96–5.70	**<0.001**	4.07	2.15–7.70	**<0.001**
Gender							
Male	280	Ref					
Female	235	0.97	0.60–1.58	0.905			
Performance status							
0	432	Ref					
1–3	80	2.00	1.15–3.48	**0.014**	1.84	1.00–3.38	**0.049**
Location							
Stomach	313	Ref					
Others	202	0.88	0.53–1.46	0.632			
Tumor size (cm)							
≦10.0	350	Ref					
>10.0	164	1.77	1.09–2.88	**0.022**	1.54	0.88–2.69	0.13
Tumor rupture							
No	417	Ref					
Yes	90	2.00	1.16–3.46	**0.012**	1.56	0.81–3.00	0.18
Mitosis (/50HPF)							
≦5	271	Ref					
>5	241	1.99	1.20–3.29	**0.007**	2.66	1.47–4.80	**0.001**
Genotyping							
KIT	457	Ref					
Others	36	2.77	1.31–5.85	**0.008**	2.88	1.25–6.59	**0.013**
Histological type							
Spindle	433	Ref					
Epithelioid & Mixed	60	2.52	1.41–4.52	**0.002**	2.05	1.05–4.01	**0.035**
Duration of adjuvant (years)							
≦3	366	Ref					
>3	149	0.27	0.13–0.56	**<0.001**	0.30	0.13–0.69	**0.004**

Bold indicates the *p* values less than 0.05 were considered significant.

Multivariate Cox proportional hazards model analysis revealed that mitosis, histological type, and duration of adjuvant therapy were significantly associated with both RFS and OS.

**FIGURE 3:**
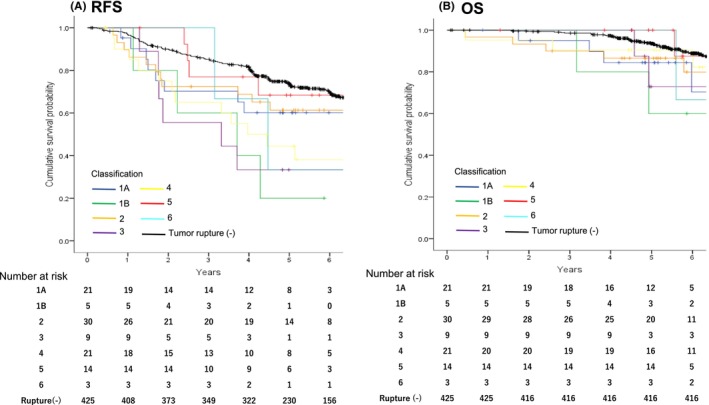
RFS and OS after surgery according to 90 patients of reevaluated report of tumor rupture based on the universal definition including non‐tumor rupture patients. A shows the RFS curves and B shows the OS curves. The blue line indicates type 1A in the universal definition, the green line indicates type 1B, the orange line indicates type 2, the purple line indicates type 3, the yellow line indicates type 4, the red line indicates type 5, the light blue line indicates type 6, and the black line indicates the non‐tumor rupture group.

Additionally, we examined prognostic outcomes of patients with each subtype of tumor rupture. As shown in Figure 3A, B, RFS of patients with any of subtypes with tumor rupture tended to have a poorer prognosis than patients without tumor rupture, however, statistical analysis is not available among each subtype due to the small number of patients.

## 5 DISCUSSION

The universal definition of tumor rupture in GIST was proposed in 2019.^15^ Using data from a prospective registry of high‐risk GISTs, we compared individual surgeon judgment of tumor rupture with those based on the universal definition. The results showed that the universal definition identified GISTs with poorer prognostic outcome. Adjuvant therapy for more than 3 years improved RFS of patients with tumor rupture. When a prospective registry study started, there was no definition of tumor rupture, hence, diagnosis of tumor rupture was left to individual investigators in the initial case reports. After the definition, tumor rupture was reevaluated based on the universal definition.

Comparing two judgments, patients with tumor rupture were almost the same for tumor fracture/spillage (type 1A/B), gastrointestinal perforation on the tumor (type 3), and piecemeal resection/intralesional dissection (type 5), suggesting that type 1A/B, type 3, and type 5 are commonly shared as tumor rupture among participating investigators. On the contrary, the number of patients for blood‐stained ascites (type 2), adjacent organ infiltration (type 4), and incisional biopsy (type 6) increased from 19 to 30, from 7 to 21, and from 0 to 3, respectively, suggesting that these three types may not be commonly recognized by surgeons as tumor rupture.

Blood‐stained ascites was defined as preoperative tumor rupture in the SSGXVIII/AIO tria.^19^ In our study, among 30 patients with blood‐stained ascites, one had gastrointestinal perforation at the tumor site, and eight had tumor fracture or spillage at laparotomy. Thus, blood‐stained ascites is considered to be tumor rupture. Incisional biopsy is technically similar to a piecemeal resection and is not recommended in oncology, rather, endoscopic or percutaneous needle biopsy is recommended as a biopsy for histological diagnosis. Histologic invasion of adjacent organs is very infrequent in GISTs. Tumor cell infiltration into adjacent organs implicates a disruption of the peritoneal lining and fibrous capsule between GISTs and neighboring organs and potential exposure of tumor cells to the peritoneal cavity. Retrospective studies have indicated that GISTs with histological invasion have worse prognostic outcomes than GISTs without it. In our study, patients with these three types of tumor rupture appear to have high recurrences similar to those with type 1A/B, type 3, and/or type 5 rupture (Figure 3). Furthermore, although the liver was the most frequent locus as the first recurrence site in the NTR group, the peritoneum was the most frequent in the TR group as indicated by previous report.^20^, ^21^ These results indicate that GISTs with any one of six rupture types may harbor potential dissemination of tumor cells in the abdominal cavity. Collectively, inclusion of blood‐stained ascites, incisional biopsy, and histologic invasion may facilitate selection of patients with worse prognostic outcomes than those with high‐risk GISTs.

The 5‐year RFS rates of patients with tumor rupture according to individual surgeons' judgment and of those based on the universal definition were 55.2% and 50.5%, respectively. Furthermore, tumor rupture based on the universal definition, but not rupture according to individual judgment, was significantly related with poorer OS than those without rupture. These results indicate that diagnosis of tumor rupture based on the definition may be preferable for selection of GIST patients with higher risk of recurrences. In this study, mitosis, histological type, and duration of adjuvant therapy are significantly prognostic for both RFS and OS of patients with high‐risk GISTs (Tables 4 and 5). Recently, several retrospective studies showed that tumor rupture was significantly associated with high recurrence rates even in the presence of 3‐year adjuvant therapy, and some indicated that tumor rupture was an independent prognostic factor for worse RFS in the era of adjuvant imatinib.^16^, ^22^, ^23^ Thus, tumor rupture is considered to be an important prognostic factor for GIST patients, especially when it is diagnosed based on the universal definition.

All clinical trials of adjuvant therapy for 1 year, 2 years, 3 years, or 5 years show improved RFS, and recurrences are infrequent during adjuvant therapy and increase after completion of adjuvant therapy.^24^, ^25^, ^26^ Recent retrospective studies have indicated that 5‐year adjuvant therapy improved RFS of patients with ruptured GISTs compared with those who underwent 3‐year adjuvant therapy.^22^, ^23^ In this study, ruptured GIST patients, who underwent adjuvant therapy for more than 3 years, showed significantly better RFS than those with adjuvant therapy for less than 1 year or those with that for 1 to 3 years (Figure 2A). These results indicate that, as in previous reports, patients that relapse after stopping adjuvant therapy generally relapse within 3 years. Therefore, patients with ruptured GISTs may be recommended longer adjuvant therapy than 3 years to suppress recurrences. In terms of OS, however, prognostic effects of adjuvant therapy are yet to be settled; OS of patients with ruptured GISTs was significantly improved by 3‐year adjuvant therapy compared with 1‐year adjuvant in the SSG XVIII/AIO study, whereas there is no difference in OS of patients with ruptured GISTs between 2‐year adjuvant therapy and no therapy in the EORTC STBSG 62024 study. In this study, we did not detect any differences in OS among three adjuvant duration (Figure 3B), probably due to short follow‐up and a small number of events. Continued adjuvant therapy may contribute to OS if it suppresses recurrence, but this study was not originally designed to examine this. Thus, it is difficult to find optimal duration of adjuvant therapy for OS.

There are several limitations in this study. There were potential biases because this study was not randomized. The results of this study were clinical outcomes without the protocol strictly regulating treatments for GIST although the guidelines were adopted by physicians in Japan.^17^ This study only included Japanese patients and was an analysis of high‐risk GIST patients. However, the results of RFS and OS were similar to previous trials conducted in the US and EU.^24^, ^25^, ^26^, ^27^ There was no statistical analysis with respect to six subtypes of tumor rupture classified according to the universal definition. However, 90 cases of tumor rupture could be accumulated, which was reliable data for the prognostic analysis of patients with ruptured GISTs. For analysis by type of tumor rupture, it is difficult to conduct a randomized controlled trial because tumor rupture itself is infrequent in a rare malignancy, and a prospective registry trial would be more feasible.

In conclusion, among 507 patients with high‐risk GISTs, 90 patients (18%) were identified as having ruptured tumors based on the universal definition. Comparing individual surgeon judgments of tumor rupture with those based on the universal definition, it was revealed that the universal definition identified GISTs with poorer prognostic outcomes. The adjuvant therapy for RFS for more than 3 years is recommended for patients with high‐risk GISTs that meet the universal definition of tumor rupture.

## FUNDING INFORMATION

The registry itself is mainly supported by Novartis Pharma. A part of this work including analysis of the STAR ReGISTry is supported in part by a grant (31‐A‐14) from the National Cancer Center Research and Development Fund and in part by a grant (19H03722 and 20K21639) for scientific research from the Japanese Ministry of Education, Culture, Sports, Science, and Technology.

## CONFLICT OF INTEREST STATEMENT

One of the co‐authors of this manuscript is Editor in Chief of *Annals of Gastroenterological Surgery*. Coauthor Y. K. is Editor in Chief of *Annals of Gastroenterological Surgery*. Coauthor M. O. was supported by lecture fees etc. from Taiho Pharmaceutical, Yakult Honsha, MSD, Ono Pharmaceutical, Nihon Servier, Bayer and Pfizer. Coauthor Y. K. was supported by lecture fees etc. from Ono Pharmaceutical, Taiho Pharmaceutical, Daiichi Sankyo, and Chugai Pharmaceutical, and by research expenses or scholarship donations (grants) from Ono Pharmaceutical, IQVIA, Astellas, Taiho Pharmaceutical, Daiichi Sankyo, Chugai Pharmaceutical, EPS, Shionogi, Nippon Zoki, Asahi Kasei and Nippon Kayaku. Coauthor H. C. was supported by research expenses or scholarship donations (grants) from Novartis.

## ETHICS STATEMENTS

Approval of the research protocol: Ethical approval was initially obtained from the institutional review board (IRB) of the National Cancer Center (No. 2016–250) and then from the IRBs of the other participating hospitals.

Informed Consent: Written informed consent was obtained from all participating patients.

Registry and the Registration No. of the study/trial: The STAR ReGISTry study was registered with the UMIN Clinical Trials Registry, number UMIN000009531.

Animal Studies: N/A.

## Supporting information


Figure S1.

Table S1.
