# Supplementary material for: Re‐appraisal of the universal definition of tumor rupture among patients with high‐risk gastrointestinal stromal tumors
Source: Ann Gastroenterol Surg. 2023 Apr 26;7(6):1021–31. doi: 10.1002/ags3.12684 (PMC10623932; doi:10.1002/ags3.12684)
Supplement: Supplementary file 1 — Figure S1. [file AGS3-7-1021-s002.pptx]

## Slide 1
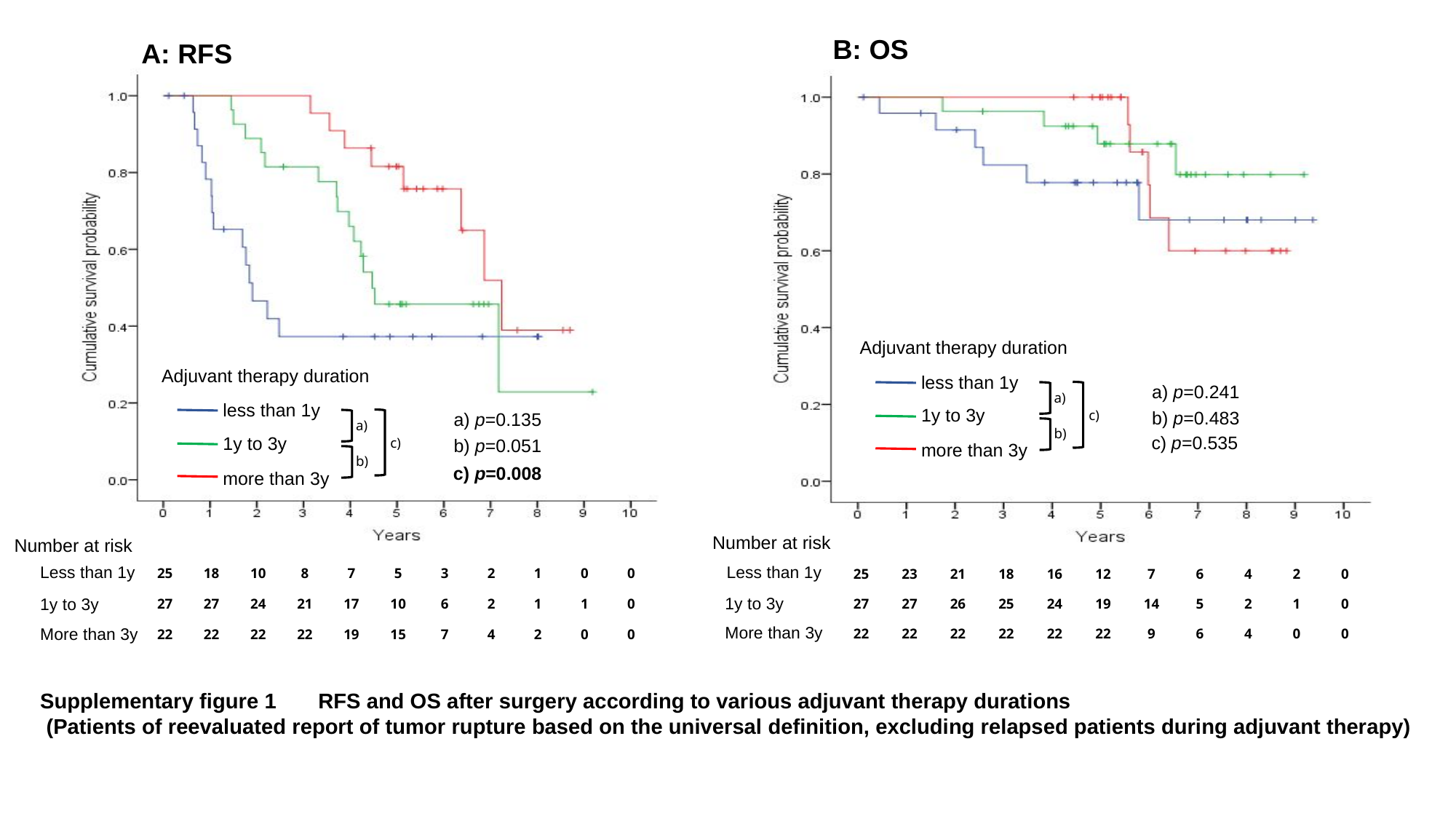

B: OS
A: RFS
Adjuvant therapy duration
Adjuvant therapy duration
less than 1y
a) p=0.241
a)
less than 1y
1y to 3y
b) p=0.483
c)
a) p=0.135
a)
b)
c) p=0.535
1y to 3y
b) p=0.051
c)
more than 3y
b)
c) p=0.008
more than 3y
Number at risk
Number at risk
Less than 1y
Less than 1y
| 25 | 18 | 10 | 8 | 7 | 5 | 3 | 2 | 1 | 0 | 0 |
| --- | --- | --- | --- | --- | --- | --- | --- | --- | --- | --- |
| 27 | 27 | 24 | 21 | 17 | 10 | 6 | 2 | 1 | 1 | 0 |
| 22 | 22 | 22 | 22 | 19 | 15 | 7 | 4 | 2 | 0 | 0 |
| 25 | 23 | 21 | 18 | 16 | 12 | 7 | 6 | 4 | 2 | 0 |
| --- | --- | --- | --- | --- | --- | --- | --- | --- | --- | --- |
| 27 | 27 | 26 | 25 | 24 | 19 | 14 | 5 | 2 | 1 | 0 |
| 22 | 22 | 22 | 22 | 22 | 22 | 9 | 6 | 4 | 0 | 0 |
1y to 3y
1y to 3y
More than 3y
More than 3y
Supplementary figure 1 RFS and OS after surgery according to various adjuvant therapy durations
 (Patients of reevaluated report of tumor rupture based on the universal definition, excluding relapsed patients during adjuvant therapy)
