# Supplementary material for: Re‐appraisal of the universal definition of tumor rupture among patients with high‐risk gastrointestinal stromal tumors
Source: Ann Gastroenterol Surg. 2023 Apr 26;7(6):1021–31. doi: 10.1002/ags3.12684 (PMC10623932; doi:10.1002/ags3.12684)
Supplement: Supplementary file 2 — Table S1. [file AGS3-7-1021-s001.docx]

Supplementary table1

The members of the STAR ReGISTry Study Group

Shizuoka General Hospital (Shinsuke Sato), Cancer Institute Hospital Japanese Foundation for Cancer Research (Masato Ozaka), Osaka Police Hospital (Yujiro Nakahara), Hokkaido University Hospital (Yoshito Komatsu), Kobe City Medical Center General Hospital (Masato Kondo), Kanagawa Cancer Center (Takanobu Yamada), Kyoto University Hospital (Yoshiharu Sakai), National Cancer Center Hospital East (Naoto Gotoda), Chiba Cancer Center Hospital (Nobuhiro Takiguchi), Hyogo Prefectural Amagasaki General Medical Center (Akina Shinkura), Kumamoto University Hospital (Hideo Baba), Juntendo University
Shizuoka Hospital (Tomoaki Ito), Niigata Cancer Center Hospital (Hiroshi Yabusaki), Yokohama Rosai Hospital (Gaku Chiguchi), Kyoto Katsura Hospital (Dai Manaka), Tokai University Hospital (Kazuhito Nabeshima), Asahikawa-Kosei General Hospital (Hiromitsu Akabane), Obihiro-Kosei General Hospital (Koichi Ono), Keio University Hospital (Norihito Wada), Toyama Prefectural Central Hospital (Masahide Kaji), Gifu University Hospital (Kazuhiro Yoshida), Matsuyama Red Cross Hospital (Kazuhito Minami), Osaka General Medical Center (Kazumasa Fujitani), Nara Medical University Hospital (Sohei atsumoto),
Osaka City General Hospital (Yutaka Tamamori), Tottori University Hospital (Yoshiyuki Fujiwara), Kitano Hospital The Tazuke Kofukai Medical Research Institute (Shugo Ueda), Kawasaki Medical School Hospital (Masahiro Yamamura), Japanese Red Cross Kumamoto Hospital (Eiji Tanaka), Jichi Medical University Hospital (Hirofumi Fujii), Yamaguchi University Hospital (Shigeru Takeda), St. Luke's International Hospital (Akihiro Suzuki), University Hospital Kyoto Prefectural University of Medicine (Eigo Otsuji), Tokyo Medical University Hachioji Medical Center (Shigeyuki Kawachi), Osaka University Hospital (Tsuyoshi Takahashi), Osaka City University Hospital (Kazuya Muguruma), Niigata University Medical and Dental Hospital (Suguru Ishikawa), Sanda City Hospital (Masaaki Mitsutsuji), Saiseikai Kumamoto Hospital (Hiroshi Takamori), Kimitsu Chuo Hospital (Takashi Kaiho), Hitachi General Hospital (Akihiro Sako), Aichi Cancer Center Hospital (Seiji Ito), National Hospital Organization Chiba Medical Center (Masahiro Mori), Center Hospital of the National Center for Global Health and Medicine (Makoto Tokuhara), University of Yamanashi Hospital (Yoshihiko Kawaguchi), Hiroshima City Asa Citizens Hospital (Jun Hihara), Ehime University Hospital (Motohira Yoshida), Yokohama Municipal Citizen's Hospital (Masazumi Takahashi), Hyogo Prefectural Kakogawa Medical Center (Shiro Takase), Kitasato University East Hospital (Keishi Yamashita), Tokyo Metropolitan Cancer and Infectious Diseases Center Komagome Hospital (Haruhiko Cho), Kizawa Memorial Hospital (Kazuya Yamaguchi), Keiyukai Sapporo Hospital (Yasunori Nishida), Sakai City Medical Center (Junya Fujita), Iwate Medical University (Keisuke Koeda), Kaizuka City Hospital (Osamu Takayama), Japanese Red Cross Nagoya Daini Hospital (Hiroshi Kanie), National Hospital Organization Shikoku Cancer Center (Shinji Hato), Seichokai Fuchu Hospital (Toshiki Hirakawa), Sendai Open Hospital (Masaya Oikawa), National Hospital Organization Kure Medical Center and Chugoku Cancer Center (Hirotaka Tashiro), Kobe University Hospital (Yoshihiro Kakeji), National Hospital Organization Kyoto Medical Center (Hiroaki Hata), Toyama University Hospital (Shinya Kajiura), Sapporo Medical University Hospital (Takayuki Nobuoka), Teikyo University Hospital (Ryoji Fukushima), South Miyagi Medical Center (Katsuro Sugiyama), Osaka Rosai Hospital (Ryohei Kawabata), Tenri Hospital (Yoshio Kadokawa), National Cancer Center Hospital (Atsuo Takashima), Yokohama City University Medical Center (Chikara Kunisaki), Saiseikai Utsunomiya Hospital (Hiroharu Shinozaki), Steel Memorial Muroran Hospital (Naoto Senmaru), Toyonaka Municipal Hospital (Hiroshi Imamura), Oita University Hospital (Satoshi Otsu), Nagoya University Hospital (Dai Shimizu), Matsushita Memorial Hospital (Akinori Noguchi), Hakodate Goryoukaku Hospital (Akinori Takagane), Tokyo Women's Medical University Yachiyo Medical Center (Atsushi Mitsunaga), Kansai Rosai Hospital (Atsushi Takeno), Yao Municipal Hospital (Junji Kawada), Nishinomiya Municipal Central Hospital (Shinichi Adachi), Iizuka Hospital (Kiyoshi Kajiyama), Tokyo Women's Medical University Hospital (Akiko Serizawa), Tokyo Medical University Hospital (Yota Shimoda), Hiroshima University Hospital (Hideki Ohdan), National Hospital Organization Okayama Medical Center (Tomokazu Kakishita), The Jikei University School of Medicine Hospital (Norio Mimori), Yokohama City University Hospital (Yasushi Rino), Japanese Red Cross Ashikaga Hospital (Takayuki Takahashi), Chiba University Hospital (Hisahiro Matsubara), Okayama Rosai Hospital (Masahiro Ishizaki), Kansai Medical University (Tatsuya Kanbara), Ishikawa Prefectural Central Hospital (Toshikatsu Tsuji), Shimane University Hospital (Noriyuki Hirahara), National Hospital Organization Kyushu Cancer Center (Mitsuhiko Ohta), Sanjo General Hospital (Tatsuo Kanda), Kawasaki Medical School Hospital (Tomoki Yamatsuji), Hoshigaoka Medical Center (Masaru Murata), Suita Municipal Hospital (Chikara Ebisui), International University of Health and Welfare, Mita Hospital (Yoshifumi Ikeda),
Iwate Prefectural Central Hospital (Jin Tejima), Yamagata University Hospital (Osamu Hachiya), Otemae Hospital (Eiji Taniguchi), Saiseikai Nara Hospital (Seiji Terauchi), National Hospital Organization Osaka Minami Medical Center (Youichi Fujita), Nagano Municipal Hospital (Masahiro Sakon), National Hospital Organization Tokyo Medical Center (Shikou Seki), Tokushima University Hospital (Mitsuo Shimada), Hyogo College of Medicine College Hospital (Mitsuru Sasako), Naha City Hospital (Hirofumi Tomori), Rinku General Medical Center (Koichi Demura), Kawasaki Hospital (Masahiro Fujikawa), National Hospital Organization Saitama National Hospital (Hirohito Ishizuka), Kashiwara Municipal Hospital (Masanari Tendo), Saitama Medical University International Medical Center (Shinichi Sakuramoto), Shimane Prefectural Central Hospital (Akiyoshi Kanazawa), Yamagata Prefectural Central Hospital (Norimasa Fukushima), Saga-ken Medical Center Koseikan (Seiji Sato), Okayama Saiseikai General Hospital (Takaomi Takahata), Clinical Research, Institute National Hospital Organization Kyushu Medical Center (Tetsuya Kusumoto), Osaka International Cancer Institute (Takeshi Omori), Tohoku University Hospital (Masanobu Takahashi), Akita University Hospital (Hiroyuki Shibata), Kagawa Prefectural Central Hospital (Norimitsu Tanaka), Hiroshima City Hiroshima Citizens Hospital (Michihiro Ishida), Hikone Municipal Hospital (Atsushi Kawabe)
